# Cardiomyopathies and a brief insight into DOX-induced cardiomyopathy

**DOI:** 10.1186/s43044-025-00628-0

**Published:** 2025-03-10

**Authors:** Sampat Singh Tanwar, Sumeet Dwivedi, Sheema Khan, Seema Sharma

**Affiliations:** 1 Shri Vaishnav Vidyapeeth Vishwadvidyalaya, Indore, India; 2 Acropolis Institute of Pharmaceutical Education and Research, Indore, India; 3 The University of Texas Rio Grande Valley, Edinburg, US

**Keywords:** Cardiomyopathy, Phenotypes, Pathogenesis, Cardiac damage, Glutathione reductases

## Abstract

**Background:**

Cardiomyopathy is a heterogeneous group of myocardial disorders characterized by structural and functional abnormalities of the heart muscle. It is classified into primary (genetic, mixed, or acquired) and secondary categories, resulting in various phenotypes including dilated, hypertrophic, and restrictive patterns. Hypertrophic cardiomyopathy, the most common primary form, can cause exertional dyspnea, presyncope, and sudden cardiac death. Dilated cardiomyopathy typically presents with heart failure symptoms, while restrictive cardiomyopathy is rarer and often associated with systemic diseases. Diagnosis involves a comprehensive evaluation including history, physical examination, electrocardiography, and echocardiography. Treatment options range from pharmacotherapy and lifestyle modifications to implantable cardioverter-defibrillators and heart transplantation in refractory cases.

**Main body:**

Anthracyclines, particularly doxorubicin, have emerged as crucial components in cancer treatment, demonstrating significant antitumor activity across various malignancies. These drugs have become standard in numerous chemotherapy regimens, improving patient outcomes. However, their use is associated with severe cardiotoxicity, including cardiomyopathy and heart failure. The mechanisms of anthracycline action and toxicity are complex, involving DNA damage, iron-mediated free radical production, and disruption of cardiovascular homeostasis. Doxorubicin-induced cardiomyopathy (DIC) is a severe complication of cancer treatment with a poor prognosis and limited effective treatments. The pathophysiology of DIC involves multiple mechanisms, including oxidative stress, inflammation, mitochondrial damage, and calcium homeostasis disorder. Despite extensive research, no effective treatment for established DIC is currently available. Dexrazoxane is the only FDA-approved protective agent, but it has limitations. Recent studies have explored various potential therapeutic approaches, including natural drugs, endogenous substances, new dosage forms, and herbal medicines. However, the lack of experimental models incorporating pre-existing cancer limits the understanding of DIC pathophysiology and treatment efficacy.

**Conclusion:**

Cardiomyopathy, whether primary or secondary, poses a significant clinical challenge due to its varying etiologies and poor prognosis in advanced stages. Anthracycline-induced cardiomyopathy is a severe complication of chemotherapy, with doxorubicin being a notable contributor. Despite advancements in cancer therapies, the cardiotoxic effects of anthracyclines necessitate further investigation into effective preventive strategies and therapeutic interventions to improve patient outcomes.

## Background

Cardiomyopathy is a heart muscle disorder characterized by abnormal structure or function, leading to progressive heart failure and potential complications. Cardiomyopathies can be primary (genetic, mixed, or acquired) or secondary (infiltrative, toxic, inflammatory). While often asymptomatic in early stages, symptoms may include shortness of breath, fatigue, cough, and edema. Diagnostic studies include B-type natriuretic peptide levels, serum chemistries, electrocardiography, and echocardiography. Treatment aims to relieve heart failure symptoms and reduce hospitalization and mortality rates, involving pharmacotherapy, implantable cardioverter-defibrillators, cardiac resynchronization therapy, and heart transplantation [1]. Cardiomyopathy is a significant global health concern, with varying prevalence across regions. In Africa, dilated cardiomyopathy is a major cause of heart failure, while peripartum cardiomyopathy is widespread [2]. Japan reports lower prevalence of dilated cardiomyopathy compared to Western populations, but similar rates for hypertrophic cardiomyopathy [3]. A French study estimated the hospital-based prevalence of cardiomyopathy at 809 per million inhabitants annually, with dilated cardiomyopathy being the most common subtype. Cardiomyopathy patients account for a substantial proportion of heart transplants and defibrillator implantations, particularly in younger individuals. The global burden of cardiomyopathy affects all ages, sexes, and ethnic groups. While invasive therapeutic procedures for cardiomyopathy have increased, in-hospital mortality has decreased [4]. These findings underscore the need for further research and targeted public health interventions to address this complex cardiac disorder. The American Heart Association classifies it into hypertrophic, dilated, restrictive, and arrhythmogenic right ventricular cardiomyopathy (ARVC). Dilated cardiomyopathy (DCM) is the most common, marked by ventricular enlargement and systolic dysfunction, often presenting with dyspnea. Hypertrophic cardiomyopathy (HCM) involves unexplained left ventricular hypertrophy due to sarcomeric gene mutations and is a leading cause of sudden cardiac death in young individuals. Restrictive cardiomyopathy (RCM) presents with impaired ventricular filling and diastolic dysfunction. ARVC features fibrofatty replacement in the right ventricle and poses risks of arrhythmias and sudden death, particularly in young males. Drug-induced cardiomyopathy is a potentially reversible form of heart disease caused by various medications and substances [5]. Common culprits include anticancer drugs, antiretroviral therapies, and certain oral antidiabetics. The pathophysiological mechanisms underlying toxic cardiomyopathy are diverse and include direct toxic effects, neurohormonal activation, altered calcium homeostasis, and oxidative stress [6]. Mitochondrial dysfunction plays a crucial role in drug-induced cardiotoxicity, leading to impaired energy production and cell death [7]. Other mechanisms involve interference with myocardial cell bioenergetics, generation of reactive oxygen species, and induction of apoptosis [8].

Doxorubicin, an effective anticancer drug, is associated with dose-dependent cardiotoxicity, primarily manifesting as cardiomyopathy. The mechanisms underlying doxorubicin-induced cardiomyopathy involve oxidative stress, mitochondrial damage, and cell death [9]. Studies have shown that cardiotoxicity can occur at doses as low as 232 mg/m^2^. At 356–388 mg/m^2^, left ventricular diastolic dysfunction develops, while at 533 mg/m^2^, anthracycline dilated myocardiopathy occurs in all cases [10]. Patients receiving ≥ 430 mg/m^2^ showed a 46% incidence of cardiomyopathy, with those over 40 years old at higher risk [11]. Myocyte damage is observed in most patients at doses ≥ 240 mg/m^2^, with heart failure occurring at higher doses [12]. The pathogenesis of DOX-induced cardiomyopathy involves multiple mechanisms. Oxidative stress, primarily caused by reactive oxygen species generated from DOX-iron interactions, plays a crucial role [13]. Impairment of autophagic degradation and mitochondrial dysfunction are also significant factors, as evidenced by accumulation of autophagosomes and suppression of mitochondrial oxygen consumption rates in DOX-treated hearts [14]. Additionally, endothelial nitric oxide synthase (eNOS) signaling contributes to the pathological response. Diagnosis typically involves imaging techniques such as echocardiograms and cardiac resonance imaging. Prevention and treatment strategies include iron chelation with dexrazoxane and the use of liposomal DOX formulations [9]. However, delayed cardiotoxicity can occur years after treatment, necessitating long-term monitoring [13].

## Main text

### Risk factors

#### Alcohol consumption

Alcoholic cardiomyopathy (ACM) is a chronic dilated heart disease associated with long-term excessive alcohol consumption, affecting up to 40% of patients with idiopathic dilated cardiomyopathy [15]. Heavy drinking (≥ 80 g/day) over several years significantly increases the risk of ACM [16]. The pathophysiology of ACM involves multiple mechanisms, including altered calcium homeostasis, oxidative stress, inflammation, and impaired protein synthesis. Chronic alcohol exposure leads to progressive myocyte loss, hypertrophy, and fibrosis, resulting in systolic and diastolic dysfunction. Alcohol also interferes with cardiac repair mechanisms, limiting myocyte regeneration. Treatment primarily involves alcohol abstinence or controlled drinking, along with standard heart failure management. Further research is needed to develop preventive and therapeutic strategies targeting alcohol-mediated myocyte damage and enhancing cardiac protective mechanisms [17].

### Obesity

Obesity cardiomyopathy is a cardiac disorder associated with severe and long-standing obesity, characterized by left ventricular dilation, hypertrophy, and dysfunction [18]. The pathophysiology involves direct effects of inflammatory mediators and indirect effects through obesity-induced hypertension, diabetes, and coronary artery disease [19]. Metabolic disturbances, including increased free fatty acids, insulin resistance, and elevated adipokines, contribute to myocardial remodeling and dysfunction [20]. The condition can progress to congestive heart failure and increase the risk of sudden cardiac death [18]. Diagnosis and clinical management of obesity cardiomyopathy remain challenging due to its similarity to dilated cardiomyopathy and the difficulty in isolating the effects of obesity from comorbidities [21]. As obesity rates continue to rise, understanding obesity cardiomyopathy becomes increasingly important for addressing its impact on cardiovascular health [20].

### Cigarette smoking

Cigarette smoking has been linked to cardiomyopathy through various studies. Experimental research on guinea pigs exposed to cigarette smoke showed increased heart weight and toxic changes in myocardial mitochondria, likely caused by carbon monoxide [22]. Similar findings were observed in rabbits, where smoking led to decreased respiration and phosphorylation rates in myocardial mitochondria [23]. These cellular changes, characterized as "smoke cardiomyopathy," primarily affect mitochondria and ribosomes, crucial for metabolic and respiratory functions. The damage is attributed to hypoxia induced by tobacco compounds, potentially leading to myocardial cell necrosis in cases of prolonged exposure [24]. A case–control study suggested that smoking may be a significant risk factor for idiopathic congestive cardiomyopathy, with the risk doubling for every 39 pack-years of smoking history [25]. These findings collectively highlight the detrimental effects of smoking on cardiac health.

### Sodium rich diet

Research suggests that high sodium intake can have significant effects on cardiac health, particularly in patients with cardiomyopathy. In hypertrophic cardiomyopathy patients, low salt intake was associated with increased syncope incidents, while higher intake reduced obstruction frequency [26]. However, excessive sodium consumption can lead to myocardial remodeling, including hypertrophy and fibrosis, even without changes in blood pressure [27]. High sodium diets may alter myocardial mechanical performance by affecting protein expression and calcium homeostasis [28]. In rats with myocardial infarction, high sodium intake promoted greater left ventricular myocyte hypertrophy compared to regular sodium intake [29]. While sodium restriction is often recommended for heart failure management, the evidence supporting this practice remains unclear [28]. These findings highlight the complex relationship between sodium intake and cardiac health, suggesting the need for individualized approaches in managing cardiomyopathy and heart failure.

### Hypertension

Hypertensive cardiomyopathy (HTN-CM) is a complex cardiac condition characterized by left ventricular hypertrophy (LVH) and diastolic dysfunction in patients with persistent hypertension [30]. While some researchers argue that HTN-CM is a distinct entity, others suggest it may represent the coexistence of hypertension with hypertrophic cardiomyopathy (HCM). HTN-CM primarily affects older individuals and can be difficult to distinguish from HCM, as both conditions share similar clinical and echocardiographic features. However, HTN-CM typically presents with thicker posterior walls compared to HCM [31]. Early detection of LVH in HTN-CM patients is crucial for optimal treatment and reducing the risk of sudden cardiac death. Diagnostic approaches include echocardiography and cardiac magnetic resonance imaging to differentiate HTN-CM from HCM [30].

### Neuromuscular disorders

Neuromuscular disorders (NMDs) are frequently associated with cardiomyopathies, which can manifest as the first sign of an underlying NMD [32]. Various types of cardiomyopathies, including hypertrophic, dilated, and restrictive, can occur in NMDs. Specific NMDs linked to cardiomyopathies include muscular dystrophies, myofibrillar myopathies, and metabolic myopathies [33]. Apical hypertrophic cardiomyopathy is rarely associated with NMDs, such as limb-girdle muscular dystrophy and glycogen storage disease [34]. Cardiac involvement in progressive muscular dystrophies often presents as impaired left ventricular systolic function, while Friedreich ataxia may exhibit left ventricular hypertrophy. Myotonic dystrophy and Emery-Dreifuss muscular dystrophy commonly present with conduction disturbances and tachyarrhythmia [35].

## Classification of cardiomyopathy

Cardiomyopathies are a group of cardiovascular diseases affecting the heart muscle, with various classifications proposed over time. The World Health Organization and International Society and Federation of Cardiology classification includes dilated, hypertrophic, arrhythmogenic right ventricular, restrictive, and unclassified cardiomyopathies. Recent studies suggest that traditional classification methods based on primary/mixed/acquired or genetic/non-genetic factors are insufficient for precise clinical management. Advances in genetic and molecular understanding have led to evolving classifications, with genetic testing becoming valuable for prognosis and treatment. Non-ischemic cardiomyopathies, which exclude coronary artery disease or ischemic injury, can be classified based on morphology, function, and genomics. The development of target-specific rescue strategies for inherited cardiomyopathies requires understanding mutation-specific molecular causes, which will continue to shape the classification and clinical characterization of these diseases [36].

### Hypertrophic cardiomyopathy

Hypertrophic cardiomyopathy (HCM) is a genetic heart condition characterized by abnormal thickening of the left ventricular wall, primarily affecting the ventricular septum [37]. While traditionally estimated at 1:500 prevalence, recent studies suggest HCM may be more common. The 1:500 estimate, initially proposed by the CARDIA study in 1995 and confirmed by UK Biobank data, translates to approximately 700,000 affected Americans and 15 million people worldwide [38]. However, advances in genetic testing, imaging techniques, and increased awareness have led researchers to reconsider this prevalence [39]. Most individuals with HCM remain asymptomatic, but some experience shortness of breath and chest pain. Hypertrophic cardiomyopathy (HCM) is a hereditary cardiovascular disorder with two main phenotypes: obstructive (HOCM) and non-obstructive (HNCM). Initially, HOCM was thought to account for 70% of cases and HNCM for 30% [40]. However, recent studies suggest the prevalence of obstructive HCM may vary between countries. In the UK, 68% of HCM cases were obstructive, while in Germany, it was 49% [41]. Importantly, Maron et al. [42] found that 70% of HCM patients had left ventricular outflow obstruction at rest or with exercise, suggesting HOCM is more prevalent than previously thought. Echocardiography remains the primary diagnostic tool for HCM [40], with exercise echocardiography recommended to detect obstruction in patients without resting gradients [42]. Treatment options differ for HOCM and HNCM, emphasizing the importance of accurate diagnosis [43]. Electrocardiographic (ECG) changes in hypertrophic cardiomyopathy (HCM) patients are common and can indicate disease progression. Long-term follow-up shows increased precordial QRS voltage, new P-wave mitral patterns, and pathologic Q wave changes in many patients [44]. ECG abnormalities, including left ventricular hypertrophy and repolarization changes, correlate with impaired systolic and diastolic mechanics [45]. HCM patients developing left ventricular apical aneurysms exhibit distinctive ECG changes, including increased QRS duration, QRS fragmentation, decreased QRS amplitude, and ST-segment elevation in V4-V6 [46]. Genetic mutations can influence ECG phenotypes in HCM patients. For instance, KCNQ1-H1717Q mutation carriers show prolonged QTc intervals, while MYLK2-K324E and KCNQ1-R190W carriers present with Q waves, ST depression, and T wave abnormalities. Importantly, ECG changes may precede echocardiographic and clinical manifestations, potentially serving as early diagnostic indicators for HCM. Treatment options for HCM include both medical and interventional approaches. Pharmacological interventions, such as beta-blockers, calcium channel blockers, and disopyramide, aim to reduce heart rate and ventricular contractility [47]. For patients with severe obstruction or symptoms refractory to medical therapy, invasive treatments are available. These include surgical myectomy and percutaneous septal ablation, which are now considered standard treatments for drug-resistant cases. Implantable cardiac defibrillators are recommended for patients at high risk of sudden cardiac death.

### Dilated cardiomyopathy

Dilated cardiomyopathy (DCM) is a significant cause of heart failure and the primary indication for heart transplantation globally [48]. The annual incidence of DCM is estimated at 5–8 cases per 100,000 in European and North American populations [49], with higher rates observed in boys, blacks, and infants [50]. DCM is characterized by cardiac chamber enlargement, impaired systolic function, and reduced ejection fraction [49]. The etiology of DCM is diverse, including genetic and environmental factors. Idiopathic cases account for the majority (66%), while myocarditis and neuromuscular diseases are common known causes [50]. Genetic studies have identified rare variants in DCM, with truncating variants in titin being the largest genetic cause [48]. The 5-year rate of death or transplantation is 46%, with age, heart failure status, left ventricular function, and cause of DCM as independent risk factors [50]. Compared to patients with other heart failure etiologies, DCM patients tend to be younger, more often male, and have fewer comorbidities [51]. While coronary artery disease patients are typically older and more likely to receive beta-blockers and calcium channel blockers, DCM patients often have more severely compromised left ventricular function and are more likely to receive diuretics, warfarin, and implantable cardioverter-defibrillators [52]. Dilated cardiomyopathy (DCM) is associated with various electrocardiographic (ECG) changes. Common findings include left atrial enlargement, prolonged PR interval, left bundle branch block (LBBB), and abnormal Q waves. T-wave inversion is the most prevalent ECG pattern in nonischemic DCM (). As cardiosclerosis progresses, the frequency and severity of intraventricular conduction disturbances increase. ECG changes can precede clinical symptoms, with 25% of patients showing ECG abnormalities as the first sign of DCM. Echocardiographic findings typically include increased left ventricular dimensions and reduced contractility. While some studies suggest a correlation between ECG patterns and ejection fraction, the relationship remains complex and not always statistically significant [53]. Treatment strategies for dilated cardiomyopathy (DCM) have evolved over time. Basic management includes inotropic therapy, preload and afterload reduction, with diuretics, digoxin, and ACE inhibitors as first-line treatments. Arrhythmia management and anticoagulation are also important. While general heart failure treatments apply to DCM, etiology-specific approaches should be considered [54]. In children, treatment is based on identifying cardiac pathophysiology, determining root causes for precision medicine, and applying therapies based on clinical status. Cause-specific therapies are emphasized for prevention and symptom management in pediatric cases [55]. For new-onset DCM, conventional heart failure treatment is established, but additional therapies like immunomodulatory and immunosuppressive treatments are under investigation. Heart transplantation remains an option for refractory cases.

### Restrictive cardiomyopathy

Restrictive cardiomyopathy (RCM) is characterized by impaired ventricular filling due to increased myocardial stiffness, resulting in diastolic dysfunction and elevated filling pressures [56]. Key features include non-dilated ventricles, atrial enlargement, and preserved systolic function. RCM can be idiopathic, familial, or secondary to systemic disorders, with familial cases often caused by sarcomeric gene mutations [57]. Diagnosis relies on echocardiography and cardiac magnetic resonance imaging, which reveal normal or slightly thickened ventricular walls, biatrial enlargement, and restrictive filling patterns. Late gadolinium enhancement can help identify specific RCM subtypes [58]. Treatment options are limited, with diuretics being the cornerstone of therapy. Prognosis is generally poor, and many patients require cardiac transplantation. Due to its rarity and complexity, management of RCM patients in expert centers is recommended. Restrictive cardiomyopathy (RCM) is a rare cardiac condition with poor prognosis in children. Electrocardiographic (ECG) abnormalities are present in 99% of RCM cases, with characteristic findings including biatrial enlargement and obliquely elevated ST segments with notched or biphasic late peaking T waves. Other common ECG features in RCM include atrial fibrillation, right axis deviation, and left and right atrial hypertrophy. Some patients may exhibit significant ST depression with T inversion, mimicking subendocardial ischemia [60]. P wave abnormalities are prevalent in pediatric RCM cases, with increased amplitude of P1 + P2 in lead V1 being a significant finding. These ECG changes reflect the restrictive physiology and may indicate abnormalities in ventricular muscle repolarization. The prognosis for RCM is poor, with a 3-year survival rate of only 26%. RCM has a poor prognosis, with high mortality rates and limited treatment options. Heart transplantation is often the only viable long-term solution, with heterotopic transplantation being an option for patients with elevated pulmonary pressures. For children with multi-organ failure, biventricular assist devices can serve as a bridge to orthotopic heart transplantation. Medical management focuses on relieving heart failure symptoms and may include pharmacotherapy, implantable cardioverter-defibrillators, and cardiac resynchronization therapy. Lifestyle modifications, such as restricting alcohol, losing weight, and following a low-sodium diet, are also recommended [61].

### Arrhythmogenic right ventricle cardiomyopathy

Arrhythmogenic right ventricular cardiomyopathy (ARVC) is a genetically determined heart muscle disease primarily affecting the right ventricle. It is characterized by progressive replacement of myocardium with fibrofatty tissue. ARVC has a prevalence of 1:1000 to 1:5000 in Europe and North America [62]. Clinical manifestations typically develop between the second and third decades of life, presenting as ventricular arrhythmias, heart failure, or sudden cardiac death, particularly in young athletes. Diagnosis is challenging due to the disease's heterogeneous nature and nonspecific symptoms, requiring a combination of electrocardiography, imaging techniques, and myocardial biopsy [63]. The understanding of ARVC has evolved from an electrical disorder to a genetic cardiomyopathy caused by defects in cell–cell adhesion proteins or intracellular signaling components [64]. Arrhythmogenic right ventricular cardiomyopathy (ARVC) is characterized by specific electrocardiographic (ECG) changes that reflect the disease's pathophysiology. Common ECG abnormalities include T-wave inversion in right precordial leads, epsilon waves, prolonged S-wave duration, and QRS prolongation in V1-V3. These changes are most prominent in anterior chest leads due to the right ventricle's proximity. ECG abnormalities correlate with disease extent and structural changes in the right ventricle [65]. Dynamic ECG changes occur in 23% of patients, emphasizing the importance of serial ECGs for early diagnosis. While ECGs are abnormal in 74% of ARVC patients and signal-averaged ECGs are positive in 60%, normal ECGs may still occur in mild cases. Overall, ECG remains a valuable, low-cost diagnostic tool for ARVC, although a normal ECG does not exclude the disease [66]. Diagnosis is challenging and relies on standardized criteria encompassing electrocardiographic, arrhythmic, morphofunctional, histopathologic, and genetic factors. Treatment options include beta-blockers, antiarrhythmic drugs, catheter ablation, and implantable cardioverter defibrillators (ICDs), with ICDs being the most effective safeguard against arrhythmic sudden death [67]. In advanced stages, heart failure therapy and anticoagulation may be necessary, with heart transplantation as a last resort. Animal models have been developed to study disease progression and screen potential treatments. Early detection, family screening, and risk stratification are crucial in managing ARVC [68].

## Doxorubicin-induced cardiomyopathy

**Fig.1:**
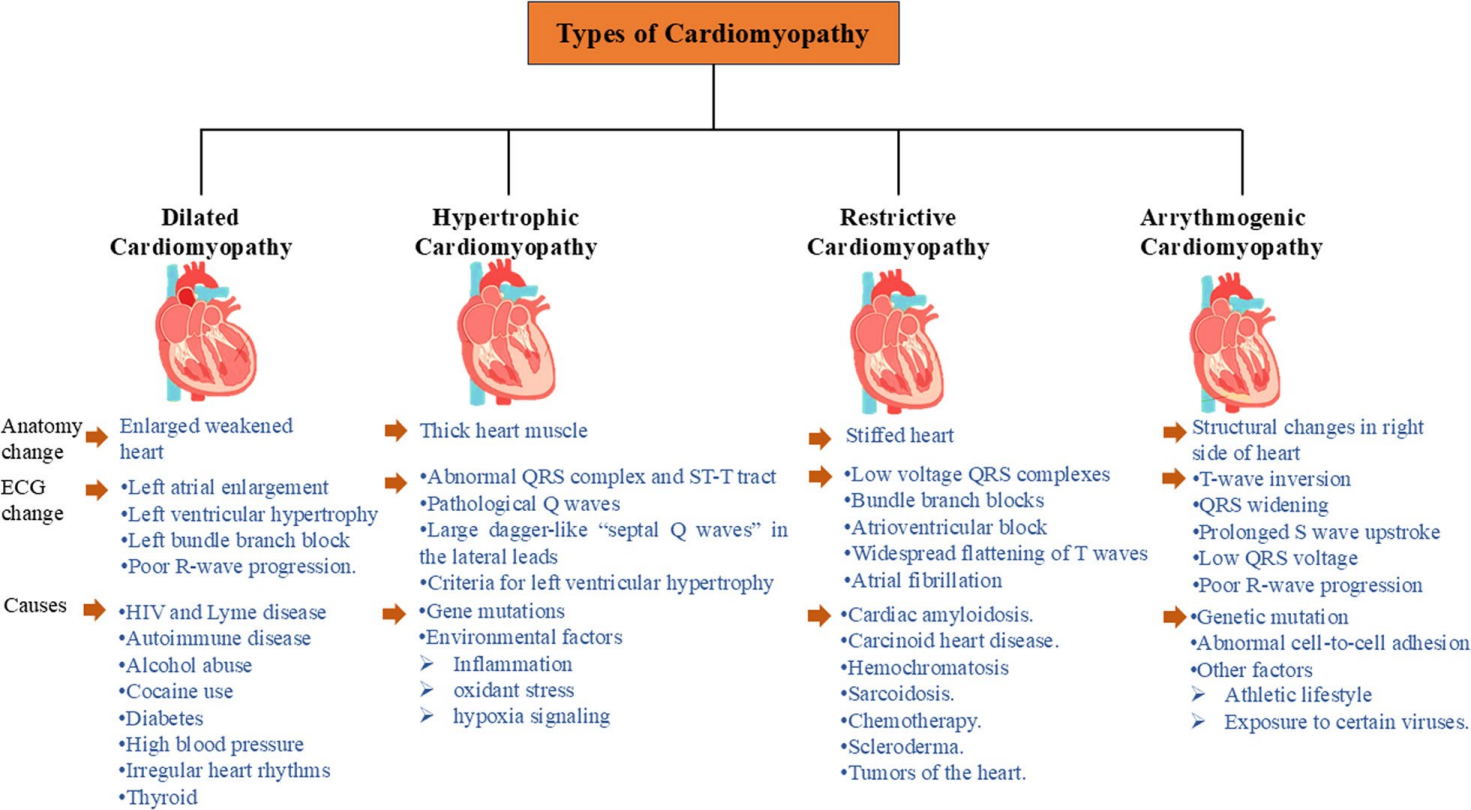
Graphical representation of types of cardiomyopathy

**Fig. 2:**
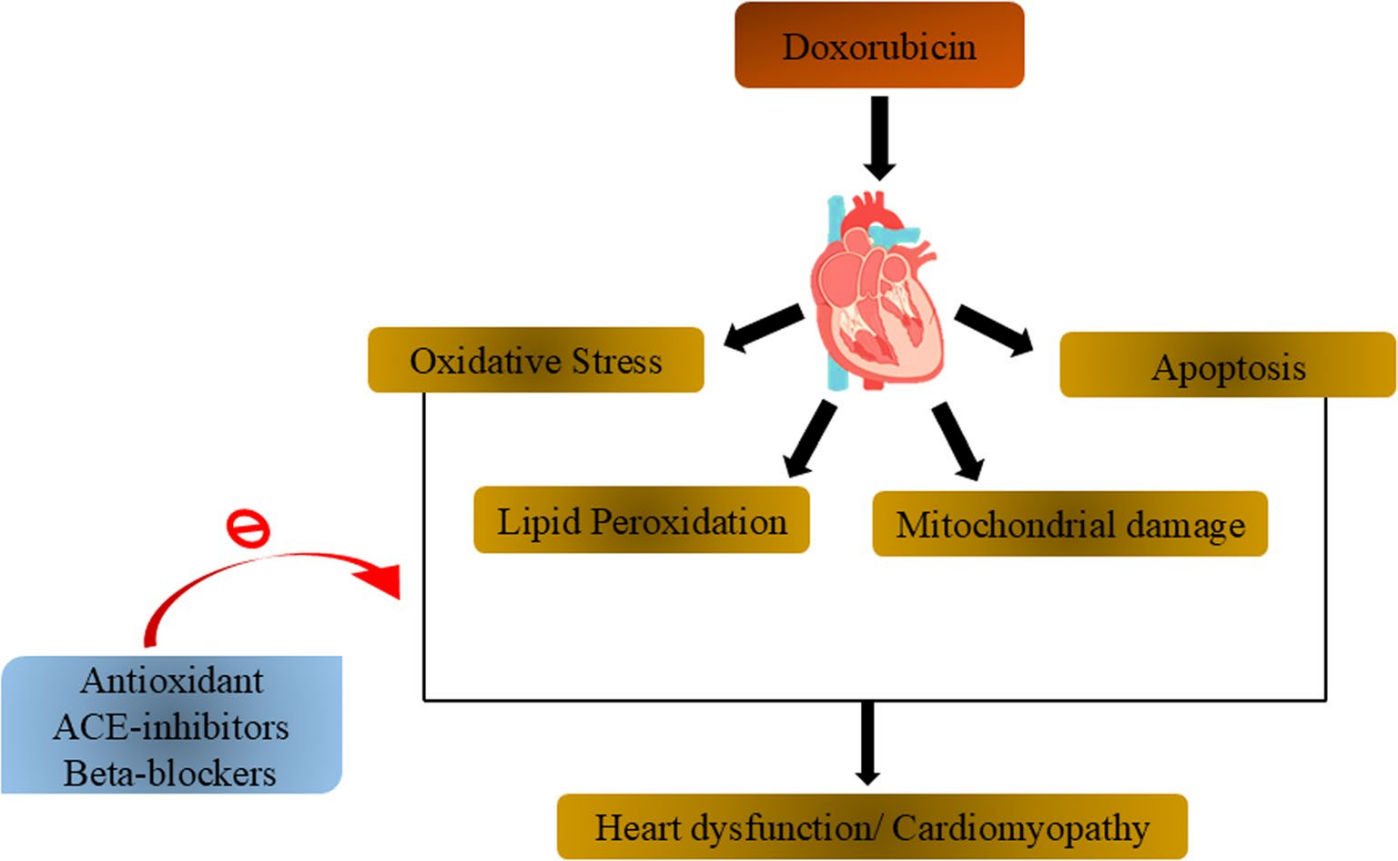
Graphical representation of DOX induced cardiomyopathy, which shows how DOX trigger different pathways and show possible treatment through Beta blockers, ACE inhibitors

**Fig. 3:**
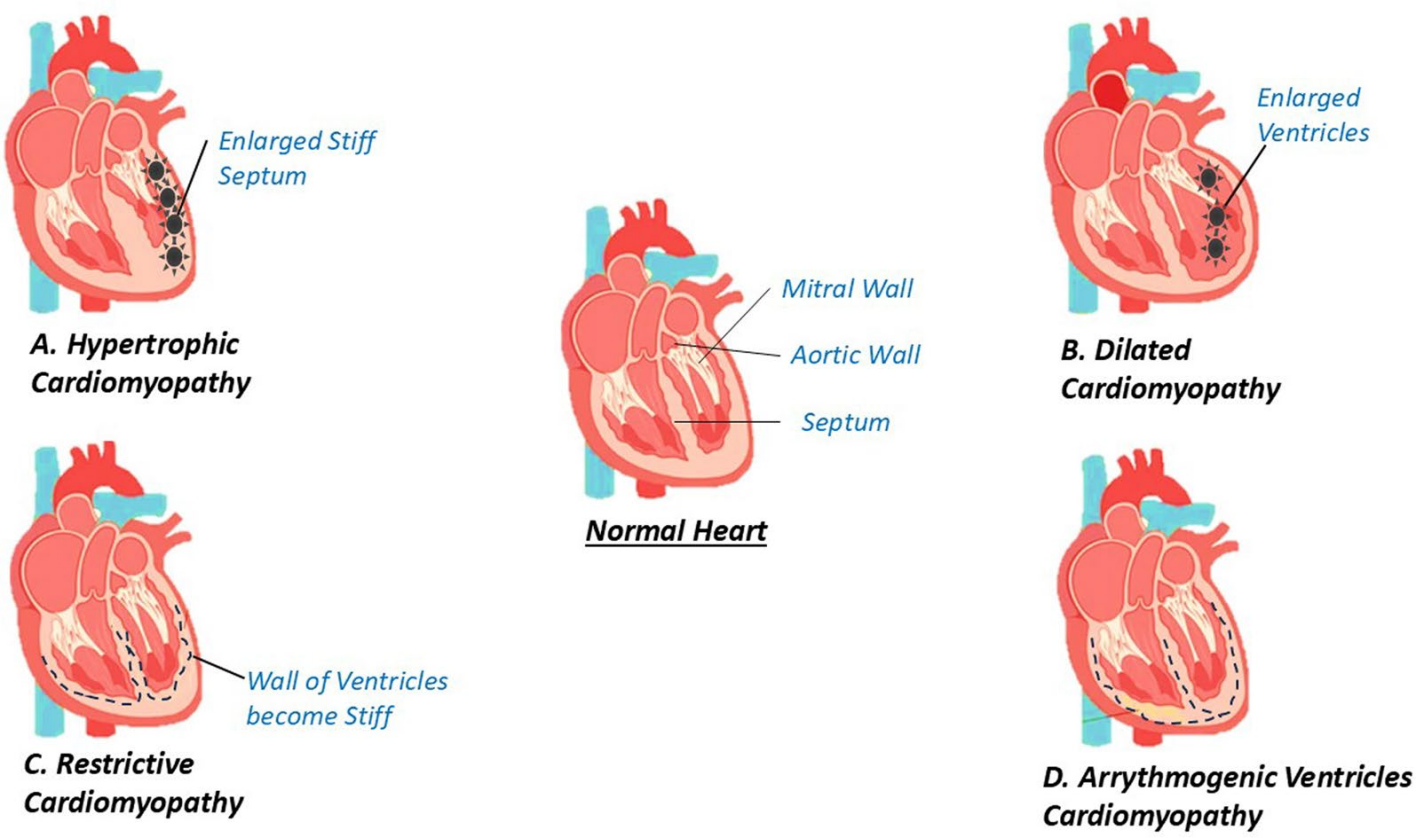
Illustrate different types of cardiomyopathies. In normal Cardiomyopathy-The walls of the heart chambers may become stretched, thickened, or stiff, and the heart may lose its ability to pump blood well **A** Hypertrophic Cardiomyopathy-It is a condition that causes the heart muscle to thicken and enlarge, making it more difficult for the heart to pump blood **B** Dilated cardiomyopathy- It is a condition in which the heart becomes enlarged and cannot pump blood effectively **C** Restrictive Cardiomyopathy-In which the walls of the heart are rigid. Thus, the heart is restricted from stretching and filling with blood properly **D** Arrythmogenic Ventricular Cardiomyopathy-In which is a heart rhythm disorder that affects the heart's ability to transmit electrical signals

Doxorubicin, an effective antineoplastic agent, is associated with dose-dependent cardiotoxicity, primarily manifesting as cardiomyopathy. The pathogenesis of doxorubicin-induced cardiomyopathy involves multiple mechanisms, with oxidative stress playing a crucial role. Mitochondrial damage occurs rapidly after doxorubicin exposure, leading to cardiac dysfunction. Other contributing factors include calcium dysregulation, endoplasmic reticulum stress, and immune system activation. Diagnosis typically involves imaging techniques such as echocardiography and cardiac MRI. Preventive strategies include the use of dexrazoxane, an iron chelator that significantly attenuates chronic cardiotoxicity [9]. Recent findings have identified a delayed form of cardiotoxicity in childhood cancer survivors, occurring up to 15 years after treatment, highlighting the need for long-term monitoring (Figs. 1, 2 and 3).

## Pathogenesis of doxorubicin-induced cardiomyopathy

### Oxidative stress

Doxorubicin (DOX) is a widely used chemotherapeutic drug that can cause severe cardiotoxicity, primarily manifesting as cardiomyopathy. While oxidative stress is a crucial factor in DOX-induced cardiomyopathy (DIC), it is not the sole mechanism [9]. Oxidative stress results from an imbalance between reactive oxygen species (ROS) and endogenous antioxidants, leading to myocardial toxicity. Several signaling pathways related to oxidative stress and inflammation play significant roles in DIC, including Nrf2/Keap1/ARE, Sirt1/p66Shc, and NLRP3/caspase-1/GSDMD [69]. Mitochondrial damage is evident shortly after DOX exposure, and chronic exposure leads to more pronounced damage. The severity of DIC is directly related to the cumulative DOX dose [9]. Doxorubicin (DOX), an anthracycline anticancer drug, exhibits dose-dependent cardiotoxicity due to its interaction with mitochondria in cardiomyocytes. The quinone moiety of DOX can be reduced to a semiquinone by mitochondrial Complex I, leading to increased reactive oxygen species (ROS) production [70]. This redox cycling generates superoxide, hydrogen peroxide, and hydroxyl radicals, overwhelming the limited antioxidant defenses of cardiomyocytes [71]. Interestingly, endothelial nitric oxide synthase (eNOS) can also reduce DOX to its semiquinone form, enhancing superoxide formation and decreasing nitric oxide production. While ROS-induced oxidative stress can lead to cardiomyocyte death, recent research suggests that DOX degradation by oxyferrous myoglobin may actually reduce its cellular toxicity. This complex interplay between DOX, mitochondria, and ROS production ultimately contributes to the development of cardiomyopathy, limiting DOX's clinical use [70]. Doxorubicin (DOX) therapy increases superoxide formation and decreases nitric oxide (NO) production due to its direct binding to the reductase domain of endothelial nitric oxide synthase (eNOS). This interaction leads to the formation of the DOX semiquinone radical and superoxide, disrupting the balance between NO and superoxide [72]. DOX-induced oxidative stress increases eNOS transcription and protein expression, further exacerbating the imbalance. The interplay between superoxide, NO, and peroxynitrite plays a crucial role in DOX-induced cell death and cardiotoxicity. Peroxynitrite, formed from the reaction of superoxide and NO, is a major trigger of DOX-induced apoptosis and necrosis in cardiac cells. Neutralizing peroxynitrite or modulating pathways leading to its generation may provide significant therapeutic benefits in mitigating DOX-induced cardiotoxicity [74].

NADPH oxidases (Noxs) play a crucial role in cardiac remodeling and hypertension through the generation of reactive oxygen species (ROS). Noxs are activated by various factors, including angiotensin II, mechanical stretch, and inflammatory cytokines. The two main cardiac isoforms, NOX2 and NOX4, have distinct effects on cardiac phenotype, with NOX2 contributing to pathological remodeling and NOX4 potentially exerting protective effects [75]. Angiotensin II regulates vascular ROS production through multiple Nox isoforms, influencing downstream signaling pathways that control cell growth, inflammation, and contraction. In the neuronal system, Nox-derived ROS are implicated in blood pressure regulation and hypertension, particularly in response to angiotensin II and aldosterone [75]. NADPH oxidases, particularly Nox2, play a significant role in doxorubicin-induced cardiomyopathy. Doxorubicin activates NADPH oxidases, leading to increased reactive oxygen species (ROS) production and subsequent cardiac apoptosis [76]. Nox2 knockout mice show attenuated cardiac dysfunction, reduced oxidative stress, and preserved sympathetic innervation compared to wild-type mice after doxorubicin treatment [77]. Nox2 deficiency also results in less myocardial atrophy, cardiomyocyte apoptosis, and interstitial fibrosis, along with reduced profibrotic gene expression and matrix metalloproteinase-9 activity. The cardiotoxic effects of doxorubicin can be mitigated by inhibiting NADPH oxidases or through treatment with the AT1 receptor antagonist losartan [78]. These findings highlight the importance of Nox2 NADPH oxidase in mediating doxorubicin-induced cardiac remodeling and dysfunction, suggesting potential therapeutic targets for preventing cardiotoxicity during cancer treatment. Nrf2 plays a crucial role in protecting against doxorubicin-induced cardiotoxicity by regulating oxidative stress and autophagy. Studies have shown that Nrf2 deficiency exacerbates doxorubicin-induced cardiac dysfunction and cardiomyocyte necrosis. Nrf2 activation has been found to ameliorate doxorubicin-induced impairment of autophagic flux and accumulation of ubiquitinated protein aggregates [80]. Various compounds, including tanshinone IIA, have demonstrated cardioprotective effects by activating Nrf2 and its downstream antioxidant genes [80]. The Nrf2/Keap1/ARE pathway, along with other signaling pathways such as Sirt1/p66Shc and NLRP3/caspase-1/GSDMD, are involved in mediating oxidative stress and inflammation in doxorubicin-induced cardiomyopathy [69]. Given its multifaceted role in cardioprotection, Nrf2 is considered a promising target for developing therapeutic strategies to mitigate doxorubicin-induced cardiotoxicity [81]. Cardiolipin (CL), a crucial mitochondrial phospholipid, plays a significant role in doxorubicin (DOX)-induced cardiomyopathy. DOX has a high affinity for CL, leading to mitochondrial accumulation and dysfunction. Interestingly, CL deficiency may provide protection against DOX-induced oxidative stress and mitochondrial damage [82]. Sex-specific differences in cardiac CL remodeling after DOX treatment have been observed, with females showing more extensive remodeling of long-chain CL species [83]. DOX-induced cardiotoxicity involves oxidative stress, mitochondrial dysfunction, and alterations in NOS and ROS pathways. Potential therapeutic strategies include the use of CL nanodisks, which confer protection against DOX-induced mitochondrial dysfunction while maintaining anti-cancer efficacy [82], and phospholipid cardioprotection [83].

Endoplasmic reticulum (ER) stress playing a crucial role [84]. Prolonged ER stress leads to cardiomyocyte apoptosis and organ dysfunction. Dox-induced ER stress activates multiple pathways, including IRE1α/ASK1/JNK and CHOP, contributing to inflammation, apoptosis, and autophagy [85]. Additionally, Dox increases mitochondrial iNOS, promoting ER stress and inflammatory responses through TLR2 activation [86]. Recent studies have explored potential therapeutic approaches targeting ER stress in Dox-induced cardiomyopathy. Empagliflozin, an anti-diabetic drug, has shown promise in mitigating Dox-induced ER stress and cardiomyocyte apoptosis [87]. Furthermore, NOS inhibitors can reduce the detrimental effects of Dox on the myocardium [86].

### Inflammation

Oxidative stress and inflammation play significant roles in DOX-induced cardiomyopathy (DIC). Studies have shown that DOX administration leads to increased production of pro-inflammatory cytokines like TNF-α and IL-6, while reducing anti-inflammatory IL-10 levels [88]. The kinin B1 receptor mediates the inflammatory response and apoptosis in DIC, as its deletion attenuates cardiac dysfunction and normalizes inflammatory markers [89]. Furthermore, endoplasmic reticulum stress promotes inflammation through iNOS/NO-induced TLR2 activation, leading to increased expression of inflammatory mediators and cell death. These inflammatory processes occur rapidly after DOX administration, with alterations in cardiac function detectable within 24 h [88]. This inflammatory response is mediated by the p38 MAPK/NF-κB pathway, which contributes to DOXO-induced cytotoxicity in cardiac cells [90]. The Wnt/β-catenin/RAS axis is also implicated in DOXO-induced cardiomyopathy, with TNF-α acting as an upstream stimulator.Inhibition of TNF-α using infliximab or rolipram has shown protective effects against DOXO-induced cardiomyopathy in animal models. Additionally, antioxidants like taurine can ameliorate DOXO-induced cardiotoxicity by reducing oxidative stress and TNF-α production [91].

Toll-like receptors (TLRs), particularly TLR2 and TLR4, play crucial roles in doxorubicin-induced cardiotoxicity and cardiomyopathy. TLR4 signaling is a key mediator of cardiac inflammation in doxorubicin-induced cardiotoxicity. While both TLR2 and TLR4 are involved in the pathogenesis of dilated cardiomyopathy, they have distinct effects. Blocking TLR2 activity reduces mortality, attenuates cardiac dysfunction, and inhibits myocardial fibrosis in doxorubicin-induced cardiomyopathy. Conversely, TLR4 activity is essential for resolving inflammation and cardiac fibrosis, as blocking TLR4 exacerbates cardiac dysfunction and fibrosis. The mechanisms underlying doxorubicin-induced cardiotoxicity involve oxidative stress and reduced antioxidant substances in cardiac tissues. Understanding the roles of TLR2 and TLR4 in doxorubicin-induced cardiac inflammation may lead to potential therapeutic strategies for managing cardiotoxicity in cancer treatment [92].

### Fibrosis

Doxorubicin (DOX)-induced cardiomyopathy involves complex mechanisms leading to cardiac fibrosis. Low-dose DOX can directly induce fibrotic changes in cardiac fibroblasts through cell death-independent pathways, promoting their transdifferentiation into myofibroblasts and increasing expression of fibrotic markers [93]. Single-cell RNA sequencing revealed that fibroblasts are the major source of extracellular matrix in DOX-induced myocardial fibrosis, with resting fibroblasts converting to matrifibrocytes and then myofibroblasts [94]. Interestingly, cardiomyocyte-specific p53 ablation does not prevent DOX-induced myocardial reactive oxygen species generation, apoptosis, or fibrosis, suggesting the involvement of p53-independent pathways [95]. AMPK plays a crucial role in regulating cardiac fibrosis and protecting against heart disease. Activation of AMPK attenuates cardiac fibrosis by inhibiting CDK2 through p21/p27 and miR-29 family pathways, as well as downregulating TGF-β1 [96]. Exercise-induced AMPK activation alleviates cardiac fibrosis by inhibiting the NADPH oxidase-ROS pathway. AMPK's cardioprotective effects extend beyond metabolic regulation, influencing fibrosis, repair, and mitochondrial function [97]. Interestingly, cardio-specific AMPK deletion leads to cardiac dysfunction, fibrosis, and mitochondrial impairment in male mice, while females remain protected. This gender-specific effect is partially mediated by estradiol signaling. Research suggests that AMP-activated protein kinase (AMPK) plays a crucial role in doxorubicin-induced cardiotoxicity. Doxorubicin inhibits AMPK activation, resulting in SIRT1 dysfunction and p53 accumulation, which contributes to cell death. Despite energy depletion, doxorubicin reduces basal phosphorylation of AMPK and its downstream target, while activating pro-survival pathways like MAPK and Akt [98]. Metformin, an antidiabetic drug, has shown potential in reducing doxorubicin cardiotoxicity and enhancing its antitumor efficacy, possibly through AMPK activation [99]. Various AMPK activators, including metformin, statins, resveratrol, and exercise, have been proposed as potential cardioprotective agents against doxorubicin-induced damage [100]. Several potential anti-fibrotic agents have shown promise in mitigating DIC. Cilostazol demonstrated antifibrotic, antioxidative, and anti-inflammatory effects by reducing perivascular fibrosis, collagen concentration, and oxidative stress markers in mice [101]. Pirfenidone and vitamin D ameliorated cardiac fibrosis by inhibiting JNK1 signaling and MCP-1 inflammatory pathways in mice with Ehrlich ascites carcinoma [102]. Anti-retroviral protease inhibitors like indinavir and ritonavir may prevent DIC by inhibiting TLR-2 and TLR-4 signaling, which are implicated in doxorubicin cardiotoxicity [103]. Additionally, a PGC-1α agonist showed therapeutic efficacy in mitigating DIC by reducing fibrosis, oxidative stress, and necroptosis markers in mice [104].

### Autophagy

Autophagy, a crucial cellular process for maintaining homeostasis, plays a significant role in cardiovascular health and disease. It contributes to cardiac ischemia, hypertension, and diabetes by interacting with reactive oxygen species in cellular organelles. While essential for heart and vessel function, defective or excessive autophagy can lead to major cardiovascular disorders such as heart failure and atherosclerosis [105]. Autophagy acts as a double-edged sword in cardiovascular disease, with both beneficial and maladaptive effects depending on the context. Recent research suggests that modulating autophagic flux could be a potential therapeutic strategy for various cardiovascular conditions, including atherosclerosis, coronary artery disease, diabetic cardiomyopathy, arrhythmia, chemotherapy-induced cardiotoxicity, and heart failure [106]. Studies have shown that Dox impairs autophagic degradation, leading to accumulation of autophagosomes and autolysosomes in cardiomyocytes. This dysregulation is associated with mitochondrial dysfunction and suppressed oxygen consumption rates [14]. Dox affects autophagy by altering expression of key regulatory proteins and transcription factors, such as LC3, p62, Beclin, mTOR, AMPK, and TFEB. The drug also compromises lysosomal function and integrity [107]. While most studies suggest Dox upregulates cardiac autophagy, contributing to toxicity, conflicting reports exist. Interestingly, the autophagic response to Dox may be species-specific, with stimulation observed in rat models and suppression in mouse models [108]. Numerous studies have shown that doxorubicin (DOX) treatment affects autophagy both in vivo and in vitro, with implications for cardiotoxicity and cancer resistance. High-dose DOX inhibits autophagic flux in cardiomyocytes, potentially contributing to necrosis. In cancer cells, autophagy modulation is being explored as a strategy to overcome DOX resistance. The role of autophagy in DOX-induced cardiotoxicity is complex, with most studies suggesting that DOX upregulates cardiac autophagy, contributing to toxicity. DOX may induce autophagy by suppressing GATA4 and/or S6K1 expression, affecting autophagy-related genes. Interestingly, the autophagic response to DOX appears to be species-specific, with stimulation observed in rat models and suppression in mouse models [109]. These findings highlight the intricate relationship between DOX treatment and autophagy, emphasizing the need for further research to elucidate its role in both therapeutic efficacy and toxicity. Recent studies have explored the role of autophagy in doxorubicin (Dox) resistance and cardiotoxicity. Contrary to the query's suggestion, these studies indicate that autophagy upregulation may actually promote cell survival and reduce Dox-induced apoptosis. In osteosarcoma cells, inhibiting autophagy with 3-methyladenine (3-MA) or Atg7 siRNA increased Dox-induced apoptosis and growth inhibition [110]. Similarly, in triple-negative breast cancer cells, combining 3-MA with Dox enhanced cytotoxicity and altered cell death mechanisms. In cardiomyocytes and a tumor-bearing mouse model, autophagy induction with rapamycin attenuated Dox-induced cardiotoxicity, improving cell viability and reducing apoptosis [111].

AMPK plays a crucial role in regulating autophagy through multiple mechanisms. Under glucose starvation, AMPK directly activates Ulk1 by phosphorylating Ser317 and Ser777, promoting autophagy initiation. Conversely, mTOR inhibits Ulk1 activation by phosphorylating Ser757, disrupting the AMPK-Ulk1 interaction [112]. AMPK also indirectly promotes autophagy by inhibiting mTORC1, a negative regulator of autophagy. Interestingly, Ulk1 phosphorylates AMPK subunits, creating a negative feedback loop to regulate autophagy termination [113]. AMPK's role in autophagy extends to mitophagy, where it induces fragmentation of damaged mitochondria and promotes autophagy machinery translocation. Additionally, AMPK regulates autophagy-related gene expression through transcription factors like FOXO3, TFEB, and BRD4 [114].

The transcription factor GATA4 plays a crucial role in protecting cardiomyocytes from doxorubicin (DOX)-induced cardiotoxicity by inhibiting autophagy and apoptosis. DOX treatment depletes GATA4 protein levels, leading to increased autophagic flux and cardiomyocyte death. GATA4 overexpression inhibits DOX-induced autophagy and reduces cardiomyocyte death by upregulating the survival factor Bcl2 and suppressing autophagy-related genes. Mice heterozygous for a null Gata4 allele show enhanced susceptibility to DOX cardiotoxicity, while genetic or pharmacologic enhancement of GATA4 prevents cardiomyocyte apoptosis and drug-induced cardiotoxicity. However, conflicting reports exist on the effects of DOX on autophagy, with some studies suggesting species-specific responses in rat and mouse models [115].

### Apoptosis

Recent research suggests that apoptosis, or programmed cell death, of cardiomyocytes plays a significant role in the development and progression of heart failure. Studies have shown that hearts from patients with end-stage cardiomyopathy exhibit increased myocyte apoptosis, which may contribute to progressive myocardial dysfunction. This process is characterized by DNA fragmentation and chromatin condensation in myocyte nuclei. The loss of viable cardiomyocytes through apoptosis is believed to be an important mechanism in the deterioration of left ventricular function in heart failure. While the exact triggers of cardiomyocyte apoptosis in failing hearts remain unclear, regional hypoxia has been proposed as a potential factor. Interestingly, despite increased expression of the anti-apoptotic protein BCL2 in failing hearts, programmed cell death still occurs, suggesting a complex regulatory process [116]. Doxorubicin (DOX), a widely used anticancer agent, induces cardiotoxicity primarily through cardiomyocyte apoptosis. Studies in rats have shown that DOX causes dose-dependent cardiomyocyte apoptosis within 24–48 h of administration, with no additive effect from repeated dosing [117]. The mechanism involves decreased expression of mitofusin 2 (Mfn2), leading to increased mitochondrial fission and reactive oxygen species (ROS) production, ultimately resulting in cardiomyocyte apoptosis [118]. While mature cardiomyocytes may be protected against DOX-induced apoptosis downstream of mitochondrial outer membrane permeabilization (MOMP), the lack of MOMP induction can alter metabolic phenotype and induce hypertrophic remodeling. DOX exposure may also increase sensitivity to future cardiomyocyte apoptosis, potentially explaining the long latency period before the onset of dilated cardiomyopathy in cancer survivors [119].

Several compounds have shown promise in mitigating DOX-induced cardiotoxicity. Rosmarinic acid alleviates cardiomyocyte apoptosis by inhibiting Fas L expression in cardiac fibroblasts [120]. Adiponectin protects against DOX-induced cardiomyopathy through anti-apoptotic effects mediated by AMPK up-regulation [121]. Cannabidiol improves cardiac function, reduces oxidative stress, and enhances mitochondrial function and biogenesis in DOX-treated mice [122]. While the long-held belief that reactive oxygen species are the primary cause of DOX cardiotoxicity is questioned, enhancing mitochondrial biogenesis is proposed as a potential strategy to prevent or mitigate DOX-induced cardiovascular complications.

### Atrophy

Cardiac atrophy, characterized by reduced cardiomyocyte size and/or quantity, can lead to a decrease in cardiac mass. This phenomenon has been observed in various conditions, including cancer cachexia [123] and doxorubicin-induced cardiotoxicity [124]. Heterotopic heart transplantation models have demonstrated significant cardiac mass reduction, with more pronounced effects in hypertrophic hearts. Atrophic remodeling involves changes in myocardial structure, including increased capillary and myocyte densities due to cell size reduction [125]. Interestingly, despite the decrease in cardiac mass, contractile function can be preserved when normalized for cell size. Sex differences in cardiac atrophy have been reported, with males showing a more severe phenotype in cancer-induced cardiac atrophy [123]. Additionally, Dox upregulates atrogin-1, a muscle-specific ubiquitin ligase, via p38-MAPK activation in cardiac myocytes, contributing to cell size reduction [126]. The p53 and mTOR pathways play crucial roles in regulating cell growth, proliferation, and death. Activation of p53 inhibits mTOR activity through AMP kinase activation and the TSC1/TSC2 complex. mTOR, a serine/threonine kinase frequently deregulated in cancer, is involved in various cellular processes and is therapeutically targeted in cancer treatment [127].

Doxorubicin (DOX), an anthracycline chemotherapeutic, induces cardiac atrophy and dysfunction in both mice and humans. This cardiotoxicity is associated with increased expression of muscle ring finger-1 (MuRF1), a ubiquitin ligase involved in muscle atrophy. DOX activates cyclin-dependent kinase 2 (CDK2), leading to phosphorylation of forkhead box O1 (FOXO1), which upregulates MuRF1 and promotes cardiomyocyte apoptosis and atrophy [128]. Additionally, DOX increases expression of other E3 ligases, including MuRF2 and MuRF3, in high doses [129]. Exercise training has been shown to protect against DOX-induced myopathy by suppressing FOXO activity and reducing MuRF1 expression in both cardiac and skeletal muscles [130]. These findings suggest that targeting the CDK2-FOXO1-MuRF1 pathway may be a promising approach for mitigating DOX-induced cardiotoxicity. Recent studies have revealed that doxorubicin (DOX)-induced cardiotoxicity involves both cardiomyocyte apoptosis and atrophy, leading to left ventricular mass loss and cardiac dysfunction [124]. The transcription factor FOXO1 plays a crucial role in this process, as DOX exposure activates FOXO1 through cyclin-dependent kinase 2 (CDK2)-mediated phosphorylation. Activated FOXO1 upregulates pro-apoptotic genes like Bim and pro-atrophic genes such as MuRF1, contributing to cardiomyocyte death and atrophy. Inhibition of FOXO1 using small molecule inhibitors or siRNAs has shown protective effects against DOX-induced cardiotoxicity in both in vitro and in vivo models [131]. Additionally, vitamin D has been found to attenuate FOXO1-mediated atrophy gene expression in muscle cells, suggesting a potential protective role against muscle atrophy [132]. The ATP-binding cassette transporter ABCB8, located in the inner mitochondrial membrane, is essential for mitochondrial iron export and cytosolic Fe/S protein maturation. Disruption of ABCB8 results in mitochondrial iron accumulation, oxidative stress, and cardiac dysfunction. The import of ABCB8 into mitochondria is facilitated by the MIA40/ALR protein import pathway, linking mitochondrial iron homeostasis to cytosolic Fe/S cluster biogenesis. Mitochondrial iron accumulation is also implicated in doxorubicin-induced cardiotoxicity, which can be mitigated by overexpression of ABCB8 or treatment with dexrazoxane [133]. Other mitochondrial ABC transporters, such as ABCB6, ABCB7, and ABCB10, also contribute to iron metabolism and porphyrin transport, with mutations in these genes associated with various human diseases [134].

Recent research has identified several potential anti-atrophy agents to mitigate doxorubicin-induced cardiomyopathy.Enhancing mitochondrial biogenesis is proposed as a key strategy for preventing or mitigating doxorubicin-induced cardiotoxicity [135]. The NADPH oxidase 2 inhibitor GSK2795039 has demonstrated efficacy in preventing cardiac atrophy by attenuating sympathetic nerve terminal abnormalities and myocyte autophagy in doxorubicin-treated mice [77]. Additionally, inhibition of TRPC3 channels has been shown to abolish doxorubicin-induced myocardial atrophy, with the TRPC3-Nox2 complex identified as a crucial mediator of this process [136].

## Treatment of doxorubicin induced cardiomyopathy

### Pharmaceuticals

Statins have shown promise in mitigating doxorubicin-induced cardiomyopathy (DICM) through various mechanisms. Rosuvastatin protects against early stages of DICM by activating Akt signaling and preserving SERCA2-mediated calcium uptake. [137] A meta-analysis revealed that statin pretreatment significantly reduced the risk of anthracycline-induced cardiomyopathy and preserved left ventricular ejection fraction in cancer patients. [138] Fluvastatin pretreatment attenuated doxorubicin-induced cardiotoxicity in mice by reducing oxidative stress, inflammation, and apoptosis while enhancing antioxidative mechanisms. [139] Similarly, lovastatin alleviated chronic heart damage following doxorubicin treatment by counteracting cardiac stress responses, mitigating mitochondrial hyperproliferation, and protecting left ventricular posterior wall diameter [140]. Dihydropyridine calcium channel blockers have shown promise in mitigating doxorubicin-induced cardiomyopathy. Nifedipine attenuated doxorubicin-induced cardiomyocyte apoptosis by suppressing intracellular Ca2 + elevation and inhibiting the CaMKII-NF-κB pathway, leading to improved left ventricular function in mice [141]. Diltiazem demonstrated cardioprotective effects against doxorubicin-induced toxicity in rats, reducing cardiac enzyme levels and preserving normal cardiac architecture [142]. Similarly, nicardipine significantly reduced cardiac biomarkers in rats treated with hydroxy-daunorubicin, a doxorubicin analog [143]. These findings support the potential of calcium channel blockers as therapeutic agents against doxorubicin-induced cardiotoxicity. Additionally, diltiazem showed promise in reversing doxorubicin resistance in breast cancer cells by modulating gene expression, potentially enhancing the drug's anticancer efficacy while protecting the heart [144].

Ranolazine has shown promise in mitigating doxorubicin-induced cardiotoxicity and neurotoxicity. Studies have demonstrated that ranolazine protects against doxorubicin-induced oxidative stress and cardiac dysfunction by inhibiting late sodium current and reducing reactive oxygen species production [145]. In animal models, ranolazine administration following doxorubicin treatment improved left ventricular function, attenuated cardiac injury, and restored cognitive function [146]. The drug's cardioprotective effects were evidenced by normalization of fractional shortening, ejection fraction, and radial strain in echocardiographic measurements [147]. Ranolazine's mechanisms of action include reducing intracellular calcium overload, improving myocardial energetics, and suppressing inflammation in both cardiac and brain tissues. Trimetazidine (TMZ) has shown promising effects in mitigating doxorubicin (DOX)-induced cardiotoxicity and hepatotoxicity. Studies in rats demonstrated that TMZ administration significantly reduced early cardiotoxic signs and attenuated DOX-induced elevation of cardiac and hepatic enzymes [146]. TMZ also improved cardiac function, as evidenced by increased left ventricular ejection fraction and reduced histological alterations in the myocardium [148]. Furthermore, TMZ protected the myocardium from damage associated with DOX and cyclophosphamide chemotherapy, reducing the severity of fuchsinophilia and the specific area of myocardial damage [149]. The protective effects of TMZ were more pronounced with longer administration periods, with a 10-day regimen showing greater efficacy than a 5-day regimen [146].

Recent studies have demonstrated the potential of dapagliflozin, a sodium-glucose cotransporter 2 inhibitor, in mitigating doxorubicin-induced cardiotoxicity. Dapagliflozin has been shown to activate AKT/PI3K signaling, leading to reduced oxidative stress, fibrosis, hypertrophy, and inflammation in cardiac cells exposed to doxorubicin [150]. In vivo experiments revealed that dapagliflozin improved cardiac function in rats treated with doxorubicin, as evidenced by echocardiography results (1556). The protective effects of dapagliflozin include preserving ejection fraction, minimizing QRS duration increases, and reducing histopathological changes associated with doxorubicin-induced cardiotoxicity [16]. Furthermore, dapagliflozin has been found to inhibit doxorubicin-induced cardiomyocyte apoptosis in diabetic conditions by suppressing endoplasmic reticulum stress [152]. Levosimendan shows promise in mitigating doxorubicin-induced cardiomyopathy through various mechanisms. In a case report, levosimendan improved left ventricular ejection fraction from 15 to 45% in a patient with anthracycline-induced cardiomyopathy [153]. Preclinical studies demonstrate that levosimendan's cardioprotective effects are mediated by the cAMP-PKA-phospholamban pathway, highlighting the role of inotropy in doxorubicin cardiotoxicity [154]. Additionally, levosimendan attenuates myocardial apoptosis by modulating the PTEN/Akt signaling pathway [155]. The Akt/eNOS and cAMP-PKA/cGMP-PKG/PLN pathways are also implicated in levosimendan's protective mechanism [154]. Importantly, levosimendan's cardioprotective effects were observed in both sub-chronic and chronic models of doxorubicin-induced cardiotoxicity [154]. Carvedilol has shown promising results in mitigating doxorubicin-induced cardiomyopathy. Studies have demonstrated that carvedilol can protect against cardiac damage by reducing oxidative stress and preventing a decrease in left ventricular ejection fraction [156]. Early administration of carvedilol has been found to inhibit DNA damage, fibrosis, and apoptosis in cardiomyocytes while promoting cardiac stem cell markers [157]. In clinical trials, prophylactic use of carvedilol in cancer patients receiving anthracycline therapy has shown protective effects on left ventricular systolic function [158]. Carvedilol's cardioprotective effects are evidenced by preserved strain and strain-rate parameters in echocardiographic assessments [159]. However, it's important to note that while carvedilol shows promise in preventing doxorubicin-induced cardiotoxicity, it may potentially exacerbate hepatic damage [157]. Enalapril, an angiotensin-converting enzyme inhibitor, has shown potential in mitigating doxorubicin-induced cardiomyopathy. In rat models, enalapril preserved mitochondrial function, reduced free radical generation, and attenuated cardiac dysfunction associated with doxorubicin treatment [160]. Similarly, enalaprilat minimized mitochondrial damage in rats exposed to doxorubicin [161]. In a chronic mouse model, enalapril protected against cardiac dysfunction and cardiomyocyte atrophy, possibly through increased activation of the PI3K/AKT/mTOR pathway [162]. However, long-term clinical outcomes in childhood cancer survivors treated with enalapril were less promising. While initial improvements in left ventricular structure and function were observed, these benefits were transient, with deterioration occurring after 6 years of therapy [163]. Benazepril hydrochloride was found to protect against DOX cardiotoxicity by regulating the PI3K/Akt pathway and reducing apoptosis in H9c2 cells [164]. Similarly, enalapril attenuated DOX-induced cardiac dysfunction by preserving mitochondrial respiratory efficiency and reducing free radical generation in rats 167). Enalaprilat also minimized mitochondrial damage in rat hearts exposed to DOX [161]. Additionally, a modified anthracycline, N-Benzyladriamycin-14-valerate (AD 198), showed protective effects against DOX-induced cardiomyopathy in rats by attenuating molecular changes and preserving left ventricular function [165].

Recent studies suggest that sacubitril/valsartan (Sac/Val) may mitigate doxorubicin-induced cardiotoxicity. In animal models, Sac/Val demonstrated cardioprotective effects by reducing oxidative stress, inflammation, and apoptosis [166]. Sac/Val also improved cardiac function, decreased fibrosis, and promoted autophagy through the AMPKα-mTORC1 signaling pathway [167]. These effects were more pronounced with Sac/Val compared to valsartan alone. In vitro studies on cardiomyocytes showed that Sac/Val reduced doxorubicin-induced mitochondrial reactive oxygen species generation and improved cell viability [168]. A small case series of two patients with anthracycline-induced cardiomyopathy reported improved cardiac function and normalized NT-proBNP levels after Sac/Val treatment, with minimal side effects and no hospitalizations [169]. Spironolactone has shown promising effects in mitigating doxorubicin-induced cardiomyopathy. Studies in rat models demonstrate that spironolactone can prevent histological and functional alterations caused by doxorubicin, including myocardial fibrosis and elevated cardiac enzymes [170]. It also attenuates cardiac remodeling by reducing collagen deposition and cardiomyocyte apoptosis, while decreasing the expression of TGF-β1 and phosphorylated-Smad3 [171]. Spironolactone's protective effects extend to preserving cardiac function, as evidenced by improved left ventricular ejection fraction and fractional shortening in treated rats [171]. The mechanism of action is thought to involve inhibition of the renin–angiotensin–aldosterone system [172]. While these findings are promising, it's worth noting that other compounds, such as spinacetin, have also shown cardioprotective effects against doxorubicin-induced toxicity through different pathways [173]. Dexrazoxane (DEX) has been shown to effectively prevent doxorubicin (DOX)-induced cardiomyopathy in various animal models and humans [174]. DEX protects cardiac mitochondria from ultrastructural, genetic, and functional damage caused by DOX-induced reactive oxygen species [175]. The optimal DEX:DOX dose ratio ranges from 10:1 to 20:1, with administration 30 min before to 15 min after DOX [174]. DEX's cardioprotective effects are long-lasting, evident for more than 6 months after DOX treatment completion [174]. Importantly, DEX does not interfere with DOX's antitumor activity and may even enhance the effects of other chemotherapy drugs like cyclophosphamide [174]. The mechanism of DEX's cardioprotection involves iron chelation, which prevents the formation of superhydroxide radicals and subsequent mitochondrial destruction [176].

Donepezil, an acetylcholinesterase inhibitor, has shown promising results in mitigating cardiotoxicity induced by chemotherapeutic agents like doxorubicin and trastuzumab. Studies have demonstrated that donepezil attenuates left ventricular dysfunction by reducing oxidative stress, inflammation, mitochondrial damage, and multiple forms of programmed cell death, including apoptosis, necroptosis, and pyroptosis [177]. Additionally, donepezil protects against doxorubicin-induced cognitive deficits and brain pathologies without interfering with its anti-cancer efficacy [178]. While the traditional view attributes doxorubicin-induced cardiotoxicity to reactive oxygen species formation, recent research suggests that enhancing mitochondrial biogenesis may be a more effective approach to prevent or mitigate cardiovascular complications in cancer survivors [179]. Metformin has shown promising results in mitigating doxorubicin-induced cardiotoxicity. Clinical studies and animal models demonstrate that metformin co-treatment can reduce cardiac damage markers, improve heart function, and preserve cardiac tissue structure [135]. The protective effects of metformin are attributed to multiple mechanisms, including the activation of the AMPK pathway, normalization of autophagy, reduction of oxidative stress, and inhibition of cardiomyocyte apoptosis [135]. Additionally, metformin has been shown to modulate the MAPK signaling pathway, further contributing to its cardioprotective effects [180]. These findings suggest that metformin could be a valuable adjunct therapy for patients receiving doxorubicin, potentially allowing for more effective cancer treatment while minimizing cardiac side effects [181].

Recent studies have identified potential protective strategies. Oseltamivir, a neuraminidase 1 inhibitor, has shown promise in mitigating DOX-induced cardiotoxicity by suppressing Drp1-dependent mitophagy [182]. Additionally, reducing mitochondrial iron levels, either through overexpression of the iron export protein ABCB8 or administration of dexrazoxane, has demonstrated cardioprotective effects [183].

Sildenafil and other phosphodiesterase-5 inhibitors have shown promise in mitigating doxorubicin-induced cardiotoxicity. Preclinical studies demonstrated that sildenafil attenuated cardiomyocyte apoptosis, improved left ventricular function, and increased survival in doxorubicin-treated mice [184]. Similarly, tadalafil, another PDE-5 inhibitor, improved cardiac function and reduced oxidative stress without interfering with doxorubicin's anti-tumor efficacy [185]. A combination therapy of sildenafil and rapamycin showed protective effects against doxorubicin-induced cardiac and skeletal muscle injury in mice, potentially through reduction of inflammation and apoptosis [186]. However, a randomized clinical trial found no significant cardioprotective effect of sildenafil when added to doxorubicin-based chemotherapy regimens in human patients, although it was deemed safe to use [187].

Vincristine, another chemotherapy drug, has shown promise in attenuating doxorubicin-induced cardiotoxicity by activating prosurvival signals and delaying apoptosis [188]. Mitochondria-targeting small molecules have emerged as potential cardioprotective agents against doxorubicin-induced cardiac events [189]. These new insights have led to the exploration of novel therapeutic strategies, including the use of pre-existing drugs and mitochondrial-biogenesis-enhancing compounds. However, further research is needed to validate the clinical utility of these approaches and to develop effective interventions for preventing or reversing doxorubicin-induced cardiomyopathy in cancer survivors [179].

### Endogenous products

Recent studies suggest that vitamin D supplementation may mitigate doxorubicin-induced cardiotoxicity. In rat models, vitamin D improved cardiac function, reduced oxidative stress markers, and decreased inflammatory cytokines compared to doxorubicin-only treatment [69]. At the cellular level, vitamin D3 attenuated doxorubicin-induced senescence in human aortic endothelial cells by upregulating IL-10 and FOXO3a expression through the pAMPKα/SIRT1/FOXO3a signaling pathway [180]. In a mouse model of triple-negative breast cancer, vitamin D supplementation reduced doxorubicin-induced cardiotoxicity by decreasing reactive oxygen species and mitochondrial damage, without compromising doxorubicin's anticancer efficacy [190]. The protective effects of vitamin D were associated with improved cardiac antioxidant status, increased heat shock protein 20 levels, and reduced markers of oxidative stress and apoptosis [190]. Nicotinamide (NAM) and alfacalcidol (1α(OH)D3) have shown promise in attenuating cardiotoxicity by preserving calcium homeostasis, replenishing ATP stores, and inhibiting apoptotic and inflammatory pathways. Nicorandil has demonstrated efficacy in alleviating hemodynamic alterations and mitochondrial dysfunction without affecting doxorubicin's antitumor activity [191]. Nicotinamide riboside, a precursor to NAD + , has been proposed as a potential preventive and therapeutic agent for doxorubicin cardiomyopathy, although further research is needed to confirm its effectiveness [192]. Melatonin has shown promising effects in mitigating doxorubicin-induced cardiotoxicity. Studies demonstrate that melatonin suppresses oxidative stress, apoptosis, and pyroptosis in cardiomyocytes and animal models [193]. The protective mechanisms involve activation of the Sirt1/Nrf2 pathway, preservation of mitochondrial function, and modulation of cell death pathways [194]. Melatonin administration has been observed to reverse biochemical and histopathological changes caused by doxorubicin, including decreased mortality, improved body and heart weight, and reduced ascites [195]. Light and ultrastructural studies have shown that melatonin markedly suppresses doxorubicin-induced cardiomyopathy [196]. While preclinical evidence is strong, clinical studies are still limited, warranting further investigation to evaluate melatonin's efficacy in protecting against doxorubicin-induced cardiotoxicity in human patients [193]. FNDC5/irisin has shown promising cardioprotective effects in various models of cardiac dysfunction. In doxorubicin-induced cardiotoxicity, FNDC5/irisin alleviates oxidative stress and cardiomyocyte apoptosis by activating AKT/mTOR signaling and the AKT/GSK3β/FYN/Nrf2 axis [197]. It also improves mitochondrial dynamics and strengthens endogenous antioxidant defenses through AMPK-Nrf2 signaling [198]. In aging-related cardiac dysfunction, FNDC5/irisin suppresses NLRP3 inflammasome activation and cardiac inflammation by activating AMPKα [199]. Furthermore, in diabetic cardiomyopathy, FNDC5/irisin attenuates diastolic dysfunction and cardiac structural remodeling by activating integrin αVβ5-AKT signaling and reducing oxidative/nitrosative stress [200]. Interleukins play diverse roles in doxorubicin-induced cardiotoxicity. IL-10 mitigates doxorubicin-induced endoplasmic reticulum stress and apoptosis in cardiomyocytes, potentially improving cardiac function [201]. Similarly, recombinant human IL-1 receptor antagonist (rhIL-1Ra) protects against acute doxorubicin-induced cardiotoxicity in mice by reducing cardiac tissue damage and preserving cardiac function [87]. The IL-1 signaling pathway is implicated in acute doxorubicin-induced cardiotoxicity, with increased expression of IL-1β, IL-1Ra, and IL-1RI correlating with cardiac damage [87]. In contrast, IL-9 exacerbates doxorubicin-induced cardiotoxicity by promoting inflammation and apoptosis in mice. Neutralizing IL-9 with antibodies alleviates cardiac injury and dysfunction, while administration of recombinant IL-9 aggravates the damage [202]. Fibroblast growth factor-2 (FGF-2) has shown promise in mitigating doxorubicin-induced cardiomyopathy through various mechanisms. FGF-2 protects cardiomyocytes by increasing efflux transporter activity and expression via protein kinase C-dependent pathways [203]. It also activates the mTOR/Nrf-2/HO-1 pathway, reducing oxidative stress and cell death while preserving lysosomal function [204]. FGF21, a related protein, attenuates doxorubicin-induced cardiotoxicity by suppressing inflammation, oxidative stress, and apoptosis through the SIRT1/LKB1/AMPK signaling pathway. Interestingly, the low-molecular-weight isoform of FGF-2 (Lo-FGF2) appears to be more cardioprotective than the high-molecular-weight isoform (Hi-FGF2). Genetic elimination or antibody-based neutralization of Hi-FGF2 enhances cardioprotection against doxorubicin-induced damage, suggesting a potential therapeutic approach [204]. Coenzyme Q10 (CoQ10) has shown promising results in mitigating doxorubicin-induced cardiomyopathy. Studies demonstrate that CoQ10 protects against cardiotoxicity through antioxidant and anti-apoptotic mechanisms [205]. CoQ10 administration significantly improves histopathological features, reduces oxidative stress markers, and downregulates pro-apoptotic gene expression in doxorubicin-treated animal models [205]. Ultrastructural examination reveals that CoQ10 prevents mitochondrial damage and myofibrillar disarrangement caused by doxorubicin. Clinical investigations suggest that concurrent CoQ10 administration may allow for higher cumulative doses of doxorubicin (up to 900 mg/m2) without compromising its anticancer effects [206]. The cardioprotective mechanism of CoQ10 is attributed to its role as an essential component of the electron transport system and a potent intracellular antioxidant, preventing mitochondrial damage and subsequent cardiomyocyte apoptosis or necrosis [206]. Recent studies have highlighted the role of 8-oxoguanine DNA glycosylase 1 (OGG1) in mitigating doxorubicin-induced cardiomyopathy. OGG1 deficiency exacerbates cardiac dysfunction and increases mortality in doxorubicin-treated mice [207]. Conversely, cardiac-specific overexpression of OGG1 protects mitochondrial DNA and reduces cardiac fibrosis in pressure overload models [208]. The mitochondrial sirtuin SIRT3 has been shown to protect against doxorubicin-induced cardiotoxicity by maintaining OGG1 levels and preventing mitochondrial DNA damage. Nutrient deprivation in cardiomyocytes leads to impaired base-excision repair through loss of OGG1, which can be rescued by recombinant OGG1 [209].

MicroRNAs (miRNAs) play a crucial role in doxorubicin-induced cardiomyopathy (DIC) and show potential as biomarkers and therapeutic targets. miR-488-3p has been identified as a protective miRNA against DIC by inhibiting CyclinG1 expression, reducing cardiomyocyte autophagic flux blockage and apoptosis [210]. Several other miRNAs, including miR-34a, miR-187, and miR-199a, have been found to be differentially expressed in response to doxorubicin treatment [211]. Early deregulation of miRNAs such as miR-187-3p, miR-182-5p, and miR-34c-5p has been observed in human-induced pluripotent stem cell-derived cardiomyocytes exposed to doxorubicin, suggesting their potential as early cardiotoxicity biomarkers. These miRNAs are involved in multiple pathways affecting cardiomyocyte function, including oxidative stress, apoptosis, and ion channel dysfunction [212]. Osteocrin (OSTN) has emerged as a promising therapeutic agent for mitigating doxorubicin-induced cardiotoxicity and other related conditions. OSTN attenuates inflammation, oxidative stress, and apoptosis in doxorubicin-treated cardiomyocytes by activating protein kinase G (PKG) [213]. It also prevents diabetic cardiomyopathy by restoring PKG-dependent proteasomal activity [214]. While the traditional view of doxorubicin cardiotoxicity focused on reactive oxygen species, recent research suggests that enhancing mitochondrial biogenesis may be a more effective approach. OSTN's protective effects extend beyond the heart, as it ameliorates adriamycin nephropathy by inhibiting p38 mitogen-activated protein kinase through natriuretic peptide receptor-C binding [215].

Mesenchymal stem cells (MSCs) and their derived exosomes show promise in mitigating doxorubicin-induced cardiomyopathy. Studies demonstrate that adipose-derived MSCs can reduce cardiac injury markers, improve histology, and decrease apoptosis in rat models [216]. Similarly, intravenous administration of cardiac stem cell-derived exosomes improved heart function and reduced fibrosis in mice [217]. MSC therapy has been associated with improved cardiac function, reduced inflammation, and decreased myocardial fibrosis [218]. The protective mechanisms of MSCs likely involve paracrine secretion, antioxidant, and anti-inflammatory effects rather than direct differentiation into cardiomyocytes [218]. While MSC therapy shows potential for cardio-protection and regeneration, concerns remain regarding its effects on tumor progression [219]. Further research and clinical trials are necessary to determine the long-term safety and efficacy of MSC-based therapies for doxorubicin-induced cardiomyopathy [220].

## Conclusion

Recent research highlights significant gaps in understanding cardiomyopathies. For Chagas cardiomyopathy, challenges persist in elucidating pathogenesis mechanisms and improving diagnosis, particularly during the asymptomatic phase. In obstructive hypertrophic cardiomyopathy (oHCM), major evidence gaps exist in epidemiology, treatment, and disease burden, especially in European countries and Canada. While advances have been made in genetic testing and stem cell-based disease modeling for cardiomyopathies, further research is needed to refine risk assessment and patient selection for defibrillator implantation in hypertrophic, dilated, and arrhythmogenic cardiomyopathies. Current efforts focus on identifying disease-driving mechanisms and developing targeted therapies. Despite progress, cardiomyopathies remain a leading cause of heart transplantation worldwide, underscoring the need for continued research to address these knowledge gaps and improve patient outcomes.

Doxorubicin-induced cardiomyopathy (DIC) is a serious complication of cancer treatment, characterized by irreversible cardiac damage. Doxorubicin-induced cardiomyopathy (DIC) is a serious side effect of cancer treatment, affecting up to 58% of patients. The prevalence of DIC is increasing among childhood cancer survivors, with an estimated 50,000 affected by 2020. Dexrazoxane is currently the only FDA-approved protective agent for DOX cardiomyopathy, but its clinical use has limitations. Ongoing research explores various therapeutic approaches, including natural compounds, endogenous substances, and repurposed drugs, to mitigate DOX-induced cardiotoxicity. Current research focuses on clinical outcomes and cellular mechanisms of acute toxicity, but gaps exist in understanding early- and late-onset cardiotoxicity. Various pharmaceutical and non-pharmaceutical approaches have shown promise in animal models, clinical translation remains challenging due to unclear mechanisms and potential side effects. Current treatment modalities include ACE inhibitors, beta-blockers, and statins, which have shown some positive results but also have limitations. Diagnostic imaging techniques such as echocardiogram, tissue Doppler imaging, cardiac resonance imaging, and multigated acquisition are used to assess DIC. While existing pharmaceutical agents offer potential for mitigating DIC, more extensive clinical data is needed. A significant limitation in current research is the lack of experimental models using doxorubicin to treat animals with preexisting cancer, which fails to account for cancer's impact on DIC pathophysiology. Future research should consider the impact of preexisting cancer on DIC pathophysiology and treatment efficacy, as well as explore novel therapeutic approaches targeting mitochondrial dysfunction and myocardial injury.

## Data Availability

No datasets were generated or analysed during the current study.

## Abbreviations

ACM: Arrhythmogenic cardiomyopathy
DCM: Dilated cardiomyopathy
DOX: Doxorubicin
PKG: Cyclic GMP-dependent protein kinase
PTEN: Phosphatase and tensin homolog
RSV: Respiratory syncytial virus
VEGF-B: Vascular endothelial growth factor
AMPK: AMP-activated protein kinase
EndMT: Endothelial-mesenchymal transition
YAP: Yes-associated protein
DEX: Dexrazoxane
MDA: Malonaldehyde
LEVO: Levothyroxine
FOXO: Forkhead box O
AMP: Adenosine monophosphate
TLR: Toll-like receptors
ATF: Activating transcription factor
IRE: Iron responsive element
